# Effectiveness of Taijiquan in treating insomnia: A systematic review and meta-analysis of randomized controlled studies

**DOI:** 10.3389/fpsyt.2022.892453

**Published:** 2022-09-27

**Authors:** Dongmiao Han, Jinling Cheng, Jiayang Qu, Xin Wen, Xuejin Liu, Yanfeng Chen, Youliang Wen, Zicai Liu, Huiyu Liu, Ying Huang

**Affiliations:** ^1^School of Rehabilitation, Gannan Medical University, Ganzhou, China; ^2^Department of Rehabilitation Medicine, YueBei People's Hospital, Shaoguan, China

**Keywords:** Taijiquan, Tai chi, insomnia, meta-analysis, Hyposomnia

## Abstract

**Background:**

Sleep efficiency of <80% based on actigraphy was defined as insomnia as self-reported difficulty falling asleep or waking up at night three to four times per week. It is known that adequate sleep is very important for human wellbeing, affecting people's work and life, insomnia will seriously damage our daily life. There is no recognized non-drug treatment. Studies have found that Taijiquan has a positive effect on insomnia patients. This systematic review and meta-analysis will evaluate the effect of Taijiquan on insomnia.

**Methods:**

To find all randomized controlled trials exploring the effects of Taijiquan on insomnia patients in Chinese and English, eight databases (Pubmed, Embase, Cochrane library, Web of Science, CNKI, CBM, VIP, and Wanfang Data) were searched. The retrieval time is from database construction to October 2021. Searches were conducted in both English and Chinese language. A meta-analysis by mean difference (MD) and 95% confidence interval (CI) was performed with RevMan 5.3. The risk of bias for each study was accounted for according to the Cochrane Handbook. Our primary outcome was Pittsburg Sleep Quality Index. We explored sources of heterogeneity by comparing effect sizes across different types of etiology, country, control group, and intervention type. The protocol was pre-registered with PROSPERO, CRD42021284511.

**Results:**

Twenty-one RCTs published between 2004 and 2021 with 2,022 participants were included in this study. Twenty-one randomized controlled studies showed that Tai Chi significantly improved PSQI scores in patients with cancer, muscle fibrosis, and sub-health insomnia [MD = −1.16, 95% CI (−1.62, −0.71), *P* < 0.01]; There is insufficient evidence of improvement in patients with cerebrovascular disease [MD = −0.54, 95% CI (−1.58, 0.51), *P* = 0.31]; 8-form, 10-form or 24-form Yang's Taijiquan had the same effect in improving PSQI [MD = −1.33, 95% CI (−1.85, −0.81), *P* < 0.01]. When there is no treatment, exercise, exercise and health education as the control, taijiquan has a significant effect on insomnia treatment, and there is no difference in efficacy compared with cognitive behavioral therapy and health education (usual care) alone.

**Conclusions:**

The results of the study showed that Taijiquan significantly improved sleep quality in healthy adults and patients with chronic diseases, which suggests that Taijiquan may be considered as an alternative behavioral therapy in the treatment of insomnia. In the future, more high-quality, well-controlled randomized trials are needed to better inform clinical decisions.

## Introduction

Insomnia is very common in the population, which is characterized by difficulty starting or maintaining sleep, along with symptoms such as irritability or fatigue when awake (1). Adequate sleep is very important for human health, affecting people's work and life, while insomnia will seriously affect our daily life. Insomnia is defined in the fifth edition of the Diagnostic and statistical manual of mental disorders (DSM-5) as difficulty getting to sleep, staying asleep, or having non-restorative sleep despite having the adequate opportunity for sleep, together with associated impairment of daytime functioning, with symptoms being present for at least 4 weeks (2). Around the world, 15–30% of adults and 10% of adolescents suffer from some form of insomnia (3, 4). Insomnia has a great impact on the human body (1, 5, 6), first of all, insomnia will make the human immune decline, weakened resistance to various diseases. Long-term insomnia can cause high blood pressure, heart disease, high blood fat, senile dementia, and so on. Secondly, patients with long-term insomnia are easy to cause negative effects in mental aspects, such as inattention, decreased thinking ability, anxiety, depression, mental tension and other emotions, cerebral cortex dysfunction, causing plant neurological dysfunction, serious forms of psychosis, neurosis, and so on.

At present, the treatment methods for insomnia mainly include drug therapy (7), cognitive behavioral therapy (8), exercise therapy (9), mindfulness meditation (10), and traditional Chinese acupuncture therapy (11), etc. But these treatments have some limitations, no matter western medicine or Traditional Chinese medicine, the current treatment of insomnia is mainly drugs, but drugs have addictions, adverse reactions, or unstable compatibility, there are individual differences in curative effect (12) and the use of drugs like doxepin, ramelteon, and secobarbital is increasingly being discouraged due to their potential toxicity. According to the European insomnia guidelines, cognitive-behavioral therapy, which usually consists of sleep hygiene, relaxation training, sleep restriction therapy, and cognitive therapy, is the most studied non-pharmacologic treatment (13). However, it requires frequent monitoring and high maintenance costs (9). Acupuncture may be beneficial for insomnia, but it is invasive (14). Therefore, we need to find more effective, simple, and safe non-invasive treatments.

Studies have shown that Taijiquan, as a new intervention, has a good effect on insomnia. Irwin et al. (15) observed 112 healthy elderly people aged 59–86 who were randomly assigned to the Taijiquan group and the health education group, among adults with moderate sleep complaints, Taijiquan can be considered a useful non-pharmaco-logic approach to improve sleep quality in them. Siu et al. (16) studied the effect of Taijiquan or exercise on sleep of the elderly with insomnia and found that compared with the control group, the sleep efficiency of the taijiquan group was improved. However, evidence-based research remains insufficient in this area, and analysis of the efficacy of insomnia caused by different diseases is lacking. There has been no systematic review of the efficacy of various forms of Tai chi compared with different exercise interventions.

Therefore, we sought to summarize existing high-quality studies on Taijiquan intervention for insomnia, and overcome the limitations of the previous Meta-analysis, to seek a higher level of evidence-based medical evidence.

## Materials and methods

### Search strategy and selection criteria

#### Search strategy

We conducted a comprehensive search for all published and unpublished RCTs of Taijiquan for patients with insomnia or other diseases with sleep complaints, in both Chinese and English language. We searched four English databases—Pubmed, Embase, Cochrane library, Web of Science, and four Chinese databases—CNKI, CBM, VIP, and Wanfang Data, from their inception to October 20, 2021. The search strategy is outlined in Appendix 1. Using the Pubmed database as an example, the search strategy was as follows (Table 1). In addition, efforts were made to find other literature sources, such as references from all included studies and clinical trial registries, which were also searched for additional relevant studies. If the original text or relevant data is not available, we will contact the original author for more information.

**Table 1:** The specific search strategy of the Pubmed database.

**No**.	**Search items**
1	“Insomnia” [Title/abstract]
2	“Sleep disorders” [Title/abstract]
3	“Sleep disturbances” [Title/abstract]
4	“Sleep initiation” [Title/abstract]
5	“Sleep maintenance insomnia” [Title/abstract]
6	“Sleep initiation and maintenance disorders” [Title/abstract]
7	1 or 2 or 3 or 4 or 5 or 6
8	“Tai Ji” [Title/abstract]
9	“Tai-ji” [Title/abstract]
10	“Tai Chi” [Title/abstract]
11	“Taijiquan” [Title/abstract]
12	“Tai Chi Chuan” [Title/abstract]
13	“T'ai Chi” [Title/abstract]
14	“Quan, Tai Ji” [Title/abstract]
15	8 or 9 or 10 or 11 or 12 or 13 or 14
16	7 and 15

#### The inclusion criteria are presented as follows

1) Randomized and controlled design
2) Sample size ≥30
3) Duration of intervention ≥1 week
4) Use a suitable control intervention (non-active placebo or established positive control, e.g., benzodiazepine)
5) Have measurable results for sleep, any form of scale, or objective electrophysiological indicators
6) Any form of Taijiquan
7) Full text available and can get enough data.

#### Exclusion criteria

We excluded duplicate reports, reviews, conference abstracts and letters, trials enrolling patients with non-related subject research, and data that were incomplete or not obtainable. In addition, after the researchers reviewed the full text and discussed for many times, the articles identified as having serious quality defects, such as data confusion, and the articles with obvious flaws in the study design were also excluded.

### Data extraction and outcome measures

The two authors (DMH and ZCL) independently searched and screened the retrieved literature. Unqualified trials were excluded, and the differences were resolved through discussion between the two authors. For problems that cannot be solved through discussion, consult the third author (YH) jointly to assess whether the trials met the inclusion criteria.

Some baseline information is extracted from the original studies, and they include the first author and published year, sample of patients, age, sex, outcome, intervention, frequency in two groups, and follow-up period. Data are extracted independently by two investigators (DMH and ZCL), and discrepancies are resolved by consensus. We have contacted the corresponding author to obtain the data when necessary. The primary outcome is Pittsburgh Sleep Quality Index (PSQI), PSQI is the most widely used sleep assessment tool with good reliability and validity (17, 18). It has been translated and used in many countries and can accurately reflect the sleep status of patients (19).

### Assessment of risk of bias in included studies

Two review authors (DMH and ZCL) independently evaluated the risk of bias for each study according to the Cochrane Handbook for Systematic Reviews of Interventions. Disagreements were resolved by discussion or by consultation with a third reviewer (YH), when necessary. The following domains were assessed: sequence generation, allocation concealment, blinding of participants, providers and outcome assessors, completeness of outcome data, selective outcome reporting, and other sources of bias. Each potential source of bias was classified as either high, low, or unclear.

### Statistical analysis

Review Manager (RevMan) version 5.3 software was used for the meta-analysis. Since this meta-analysis included only continuous data, we used the mean difference (MD) and 95% confidence interval (CI) for analysis. If the data was not available in the article, and the authors could not be contacted, estimates were made using the known data and the formula in the Cochrane Handbook for Systematic Reviews of Interventions. The *I*^2^ statistic was used to evaluate the heterogeneity revealed by data analysis. The interpretation of the *I*^2^ statistic is as follow (Cochrane Handbook for Systematic Reviews of Interventions Version 5.0.1.2008):

- 10–40%: might not be important
- 30–60%: may represent moderate heterogeneity
- 50–90%: may represent substantial heterogeneity
- 75–100%: considerable heterogeneity.

If substantial heterogeneity (above 50%) was detected, a random-effects model was used in the meta-analysis, where appropriate, subgroup or sensitivity analyses were performed to explore the source of heterogeneity.

## Results

### Search results

**Figure 1:**
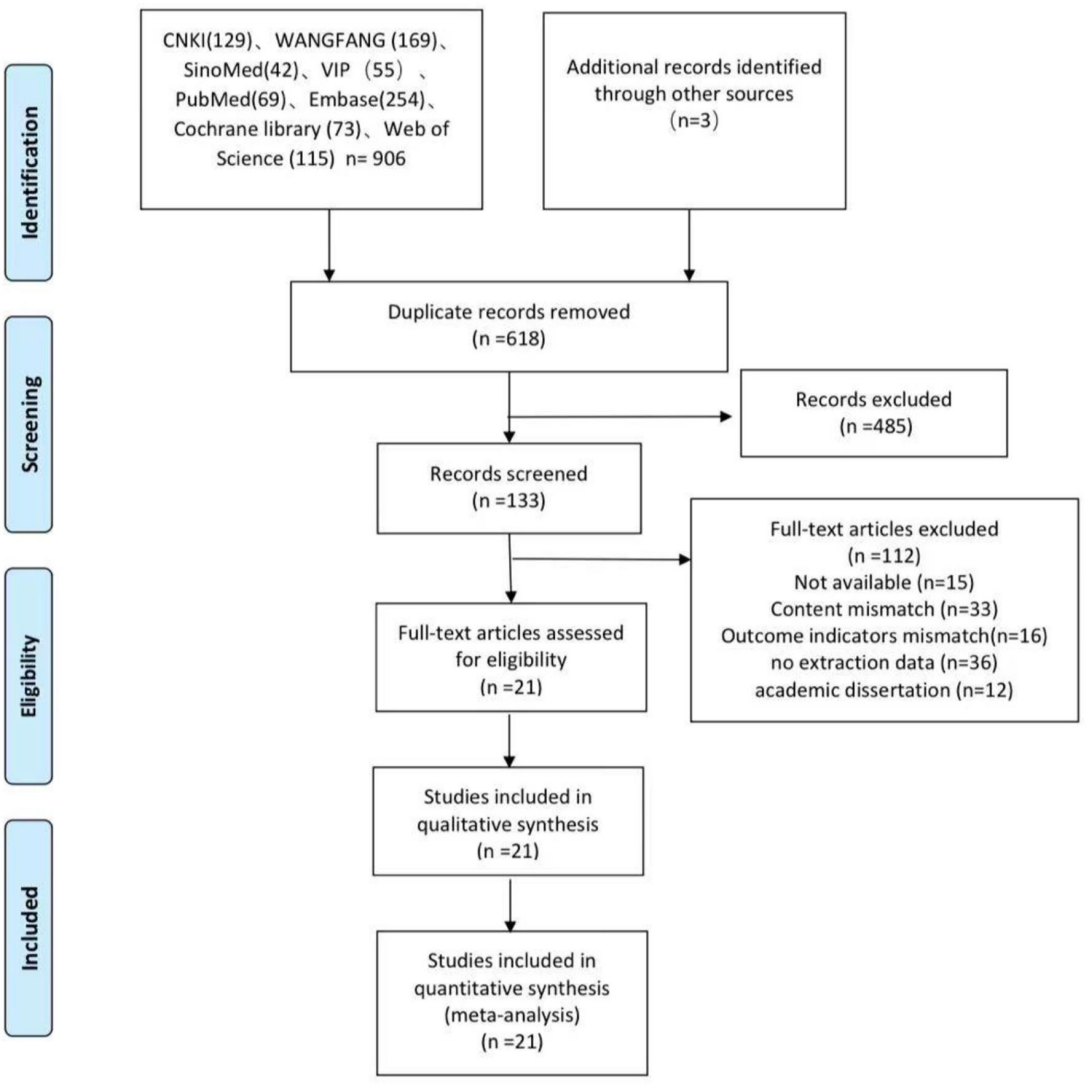
Flowchart of the study search and selection process.

The review searched literature from eight databases. Figure 1 shows the search and selection process. A total of 909 potentially relevant articles were searched for strategies. After removing the duplicates, 618 articles need to be abstracted. 485 were excluded from titles and summaries, leaving 133 requiring full text. After reading the full text of these articles, were excluded, mainly because of inadequate study design, inadequate interventions, or incomplete data. The qualitative analysis included 21 studies (15, 16, 20–38).

### Characteristics of included studies

The study included 21 articles from seven countries—four from China, 12 from the United States, one from Iran, one from Italy, one from Turkey, one from Japan, and one from Germany. Their characteristics are presented in Table 2. In all of the studies, the mean total PSQI score at baseline was ≥5. This is consistent with insomnia diagnosis. In addition to insomnia, some patients also had other diseases such as depression, prostate cancer, knee osteoarthritis, fibromyalgia, post-stroke, or breast cancer. A total of 2,022 patients were included in this meta-analysis, of which 907 were allocated to the Taijiquan group and 1,115 were allocated to the control group. The average age of patients is over 35. One article (37) did not describe gender, and there were 522 male patients and 1,456 female patients in the retained literature (Table 2).

**Table 2:** Basic information included in the study.

**Study**	**Country**	**Age (X ±S)**	**Sample (M)**	**Patient (disease)**
Siu et al. (16)	China	67.3	320 (64)	Chronic insomnia
Cheung et al. (22)	China	60.06	30 (16)	Lung cancer
Yilmaz Gokmen et al. (26)	Turkey	48.06	50 (31)	Obstructive sleep apnea
Jones et al. (30)	America	54	101 (7)	Fibromyalgia patients
Nguyen et al. (32)	Germany	68.9	96 (48)	Older people
Wang et al. (21)	America	50.1	66 (9)	Fibromyalgia patients
Irwin et al. (33)	America	65.55	123 (35)	Chronic and primary insomnia
Taylor-Piliae et al. (35)	America	69.3	28 (17)	Post-stroke with sleep complaints
Taylor-Piliae et al. (36)	America	69.9	145 (77)	Post-stroke with sleep complaints
Wang et al. (24)	America	51.8	226 (17)	Fibromyalgia
Lü et al. (29)	China	64.57	46 (0)	Knee osteoarthritis (OA)
Maddali Bongi et al. (37)	Italy	52.24	44/(N)	Fibromyalgia syndrome
Irwin et al. (15)	America	69.9	112 (41)	Healthy older adults
Zhu et al. (23)	China	35.65	80 (0)	Dependent on amphetamine-type stimulant
McQuade et al. (28)	America	64.47	66 (66)	Prostate cancer
Larkey et al. (31)	America	58.8	101 (0)	Breast cancer survivors
Frye et al. (20)	America	69.2	84 (30)	Older people
Li et al. (25)	America	75.37	118 (22)	Older adults with sleep complaints
Irwin et al. (34)	America	59.8	90 (0)	Breast cancer survivors
Wang et al. (38)	Japan	77	34 (12)	The elderly with cerebral vascular disorder
Hosseini et al. (27)	Iran.	69.1	62 (30)	Insomnia

For the interventions, 15 articles trace back or describe in detail the types of taijiquan. eight studies used 24 forms of Yang's Taijiquan, and four studies used 8-form Yang style Taijiquan. Three studies used 10-form Yang style Taijiquan. Taijiquan lasts for a minimum of 12 weeks and a maximum of 24 months. Most are 3–6 months. The frequency of Taijiquan varies from 1 to 5 times per week (Table 3).

**Table 3:** Basic information included in the study.

**Study**	**Intervention (frequency)**	**Control**	**Outcome**	**Follow-up time**
Siu et al. (16)	Yang-style 24-form Taijiquan 60 min/3*weeks	G1: Conventional exercise G2: No intervention	PSQI	24 months
Cheung et al. (22)	Yang-style 24-form Taijiquan 60 min/2*weeks	G1: Self-management group; G2: Aerobic exercise;	PSQI	1-year
Yilmaz Gokmen et al. (26)	Taijiquan 60 min/3*weeks	Home exercise	PSQI	12 weeks
Jones et al. (30)	8-form Yang style Taijiquan 90 min/twice weekly	Educational control	PSQI	24 weeks
Nguyen et al. (32)	24-form style Taijiquan 60 min/twice weekly	No intervention	PSQI	6 months
Wang et al. (21)	10-form classic yang Style 60 min/twice weekly	Education and exercises	PSQI	24 weeks
Irwin et al. (33)	Yang style 24- posture Taijiquan 120 min/once weekly	G1: Cognitive behavioral therapy; G2: Hygiene education (Sleep Seminar, SS)	PSQI	16 months
Taylor-Piliae et al. (35)	Yang style 24- posture Taijiquan 60 min/three times weekly	Exercise and usual care	PSQI	12 weeks
Taylor-Piliae et al. (36)	Yang style 24- posture Taijiquan 60 min/three times weekly	G1: Exercises; G2: Usual care	PSQI	12 weeks
Wang et al. (24)	Yang style Taijiquan 60 min/once a week	Aerobic exercise sessions	PSQI	52 weeks
Lü et al. (29)	Yang style 8-posture Taijiquan 60 min/three times weekly	Educational classes	PSQI	24 weeks
Maddali Bongi et al. (37)	Taijiquan 60 min/twice weekly	Health education and exercises	PSQI	16 weeks
Irwin et al. (15)	Taijiquan 40 min/three times weekly	Health education	PSQI	25 weeks
Zhu et al. (23)	24-form Yang style Taijiquan 60 min/times; five times /week during the first 3 months and three times a week during the second 3 months	Standard care	PSQI	6 months
McQuade et al. (28)	Yang style 8-posture Taijiquan 40 min/three times weekly	G1: No treatment; G2: Exercise	PSQI	3 months
Larkey (31)	Qigong/Taijiquan 60 min/once a week	Sham Qigong	PSQI	3 months
Frye (20)	10-form Yang style Taijiquan 60 min/three times weekly	G1: No treatment; G2: Low-impact exercises	PSQI	12 weeks
Li et al. (25)	Taijiquan-Easy Taijiquan 60 min/three times weekly	Low-impact exercise	PSQI	6 months
Irwin et al. (34)	Taijiquan 120 min/once a week	Cognitive-behavioral therapy 120 min/once weekly	PSQI	15 months
Wang et al. (38)	Classical yang Style Taijiquan 50-min Taijiquan/once a week	Rehabilitation exercises 80 min/once a week	PSQI	12 weeks
Hosseini et al. (27)	Taijiquan exercise sessions 20–25 min/three times per week	No treatment	PSQI	12 weeks

### Risk of bias in the included studies

**Figure 2:**
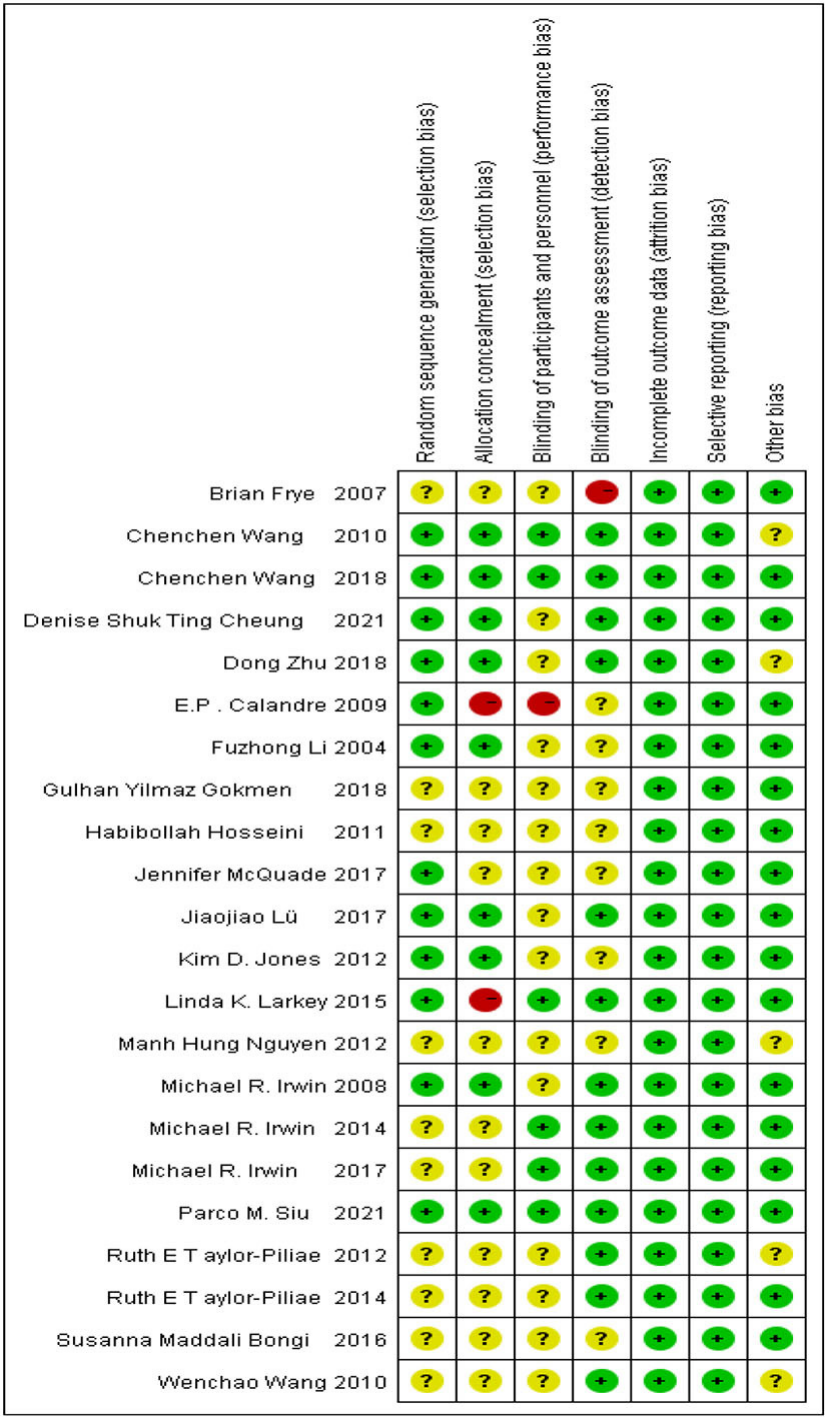
Risk of bias summary: review authors' judgments about each risk of bias item for each included study.

**Figure 3:**
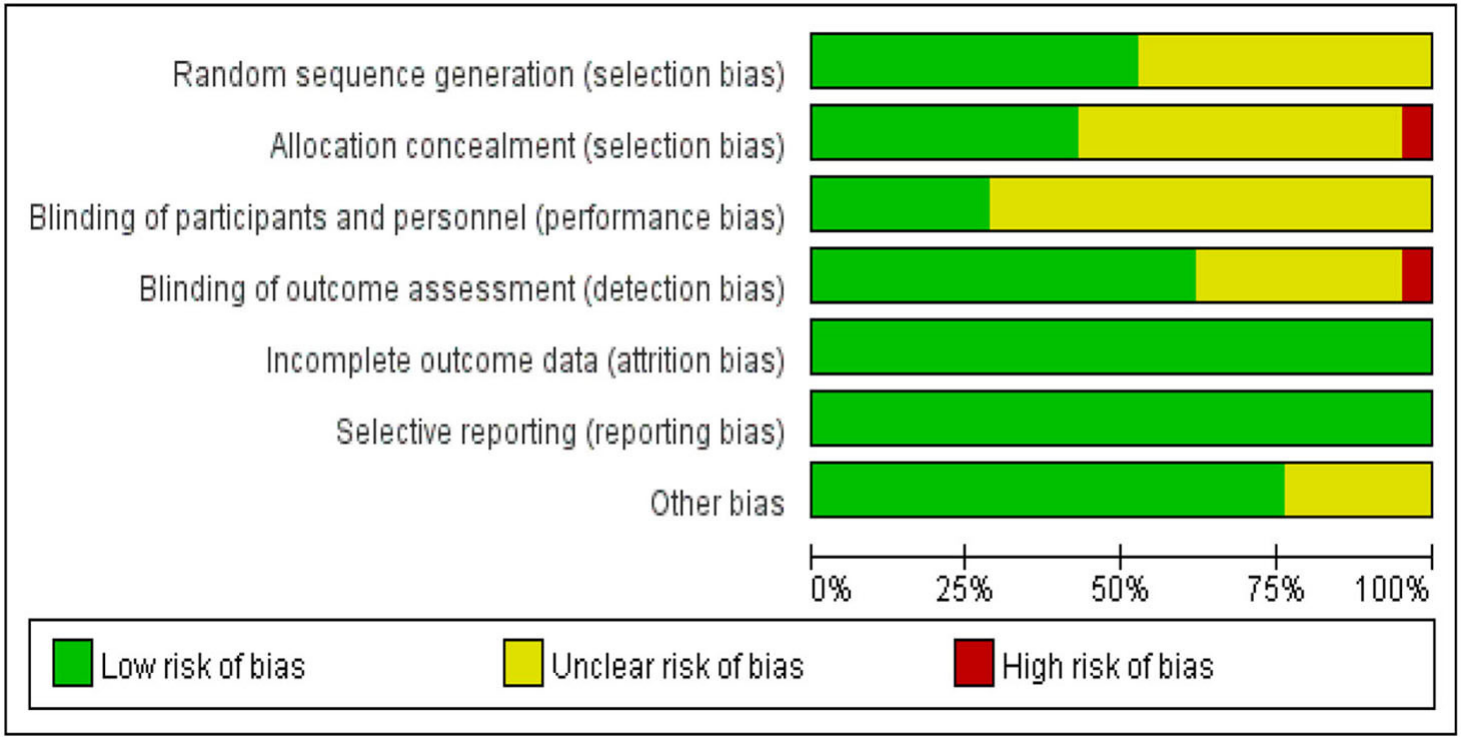
Risk of bias graph: review authors' judgments about each risk of bias item presented as percentages across all included studies.

According to the Cochrane Handbook for Systematic Reviews of Interventions, we assessed the risk of bias in the included literature. The results are shown in Figures 2, 3. 12 RCTs described the appropriate random sequence generation method in detail. The other eight studies did not report the random sequence generation. Thus, the risk for these domains was determined to be unclear. Only nine of the studies reported using allocation concealment. Only 13 reported blinding the assessors. Due to the nature of Taijiquan, strict blinding of participants was difficult. All of the included studies reported the complete outcome data, and we considered them to be low-risk for this item. Most studies reported all of the outcomes. No other significant bias was found in any of the studies.

### Meta-analysis results

According to our retrieval results and the characteristics of the included studies, most of the studies used PSQI as the outcome indicator, while other outcome indicators could not be meta-analyzed due to the lack of a sufficient number of studies.

**Figure 4:**
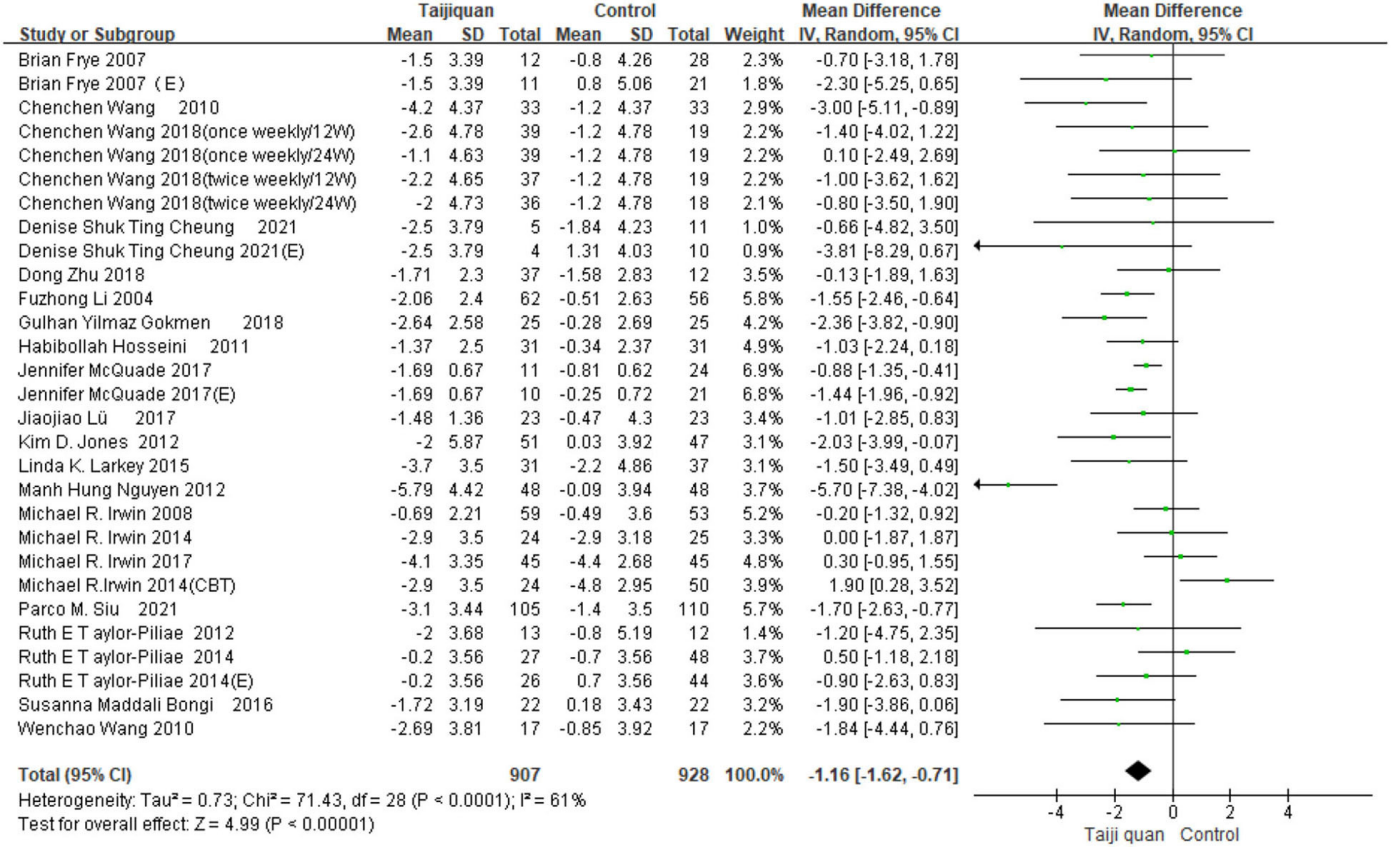
Forest plot for PSQI meta-analysis was performed on all included studies.

Our meta results suggest that Taijiquan is beneficial to insomnia patients and can significantly improve PSQI [MD = −1.16, 95% CI (−1.62, −0.71), *P* < 0.00001] (Figure 4). However, we found high heterogeneity of meta [*I*^2^ = 61%, *P* < 0.0001] (Figure 4), Further subgroup analysis is required to identify the source of heterogeneity.

**Figure 5:**
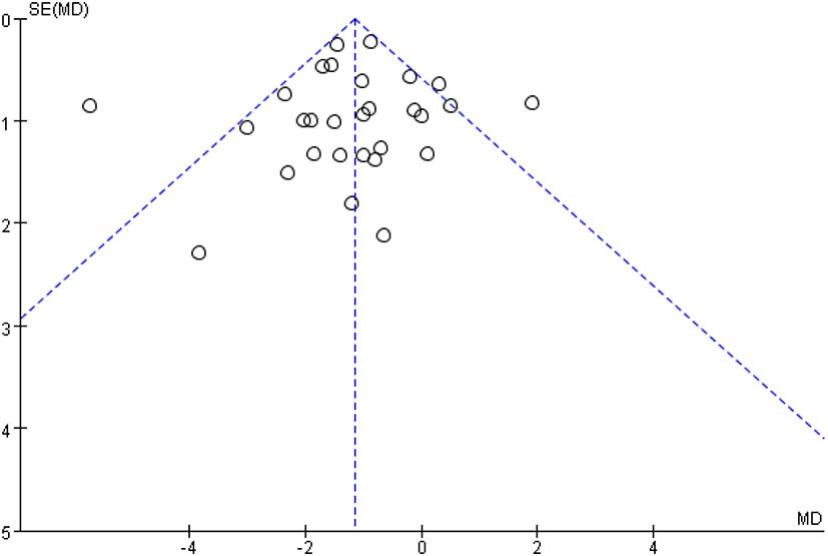
The publication bias of all studies was determined by funnel plot.

**Figure 6:**
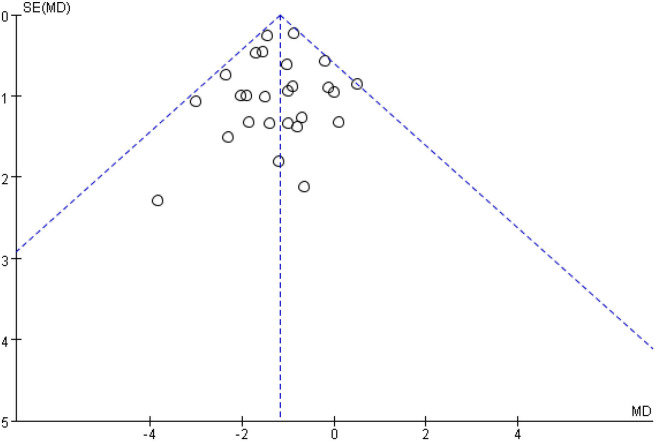
After removing three studies, the symmetrical funnel plot was obtained.

**Figure 7:**
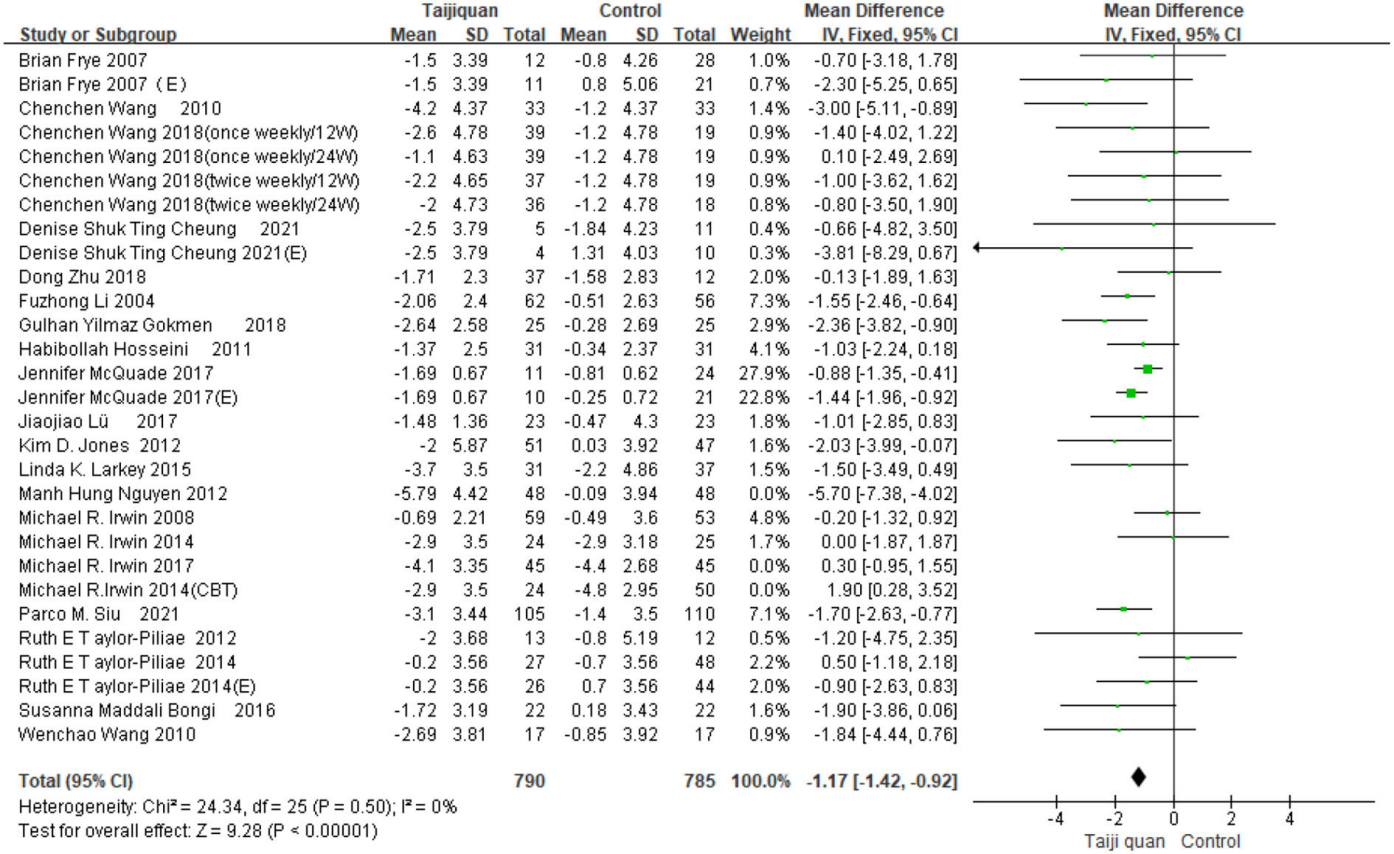
After deleting three studies, the PSQI forest plot of the remaining studies.

Figure 5 shows that the funnel diagram is symmetric, But there are three studies in the funnel outside, We use sensitivity analysis to determine that three studies with both large heterogeneity and publication bias from Irwin et al. (33, 34) studies and Nguyen et al. (32) study. After removing the three articles with publication bias, the Funnel plot is symmetric (Figure 6) and the combined results of the meta-analysis were stable [MD = −1.17, 95% CI (−1.42, −0.92), *P* < 0.00001] and the heterogeneity is small [*I*^2^ = 0, *P* = 0.05] (Figure 7).

### Subgroup analysis

**Figure 8:**
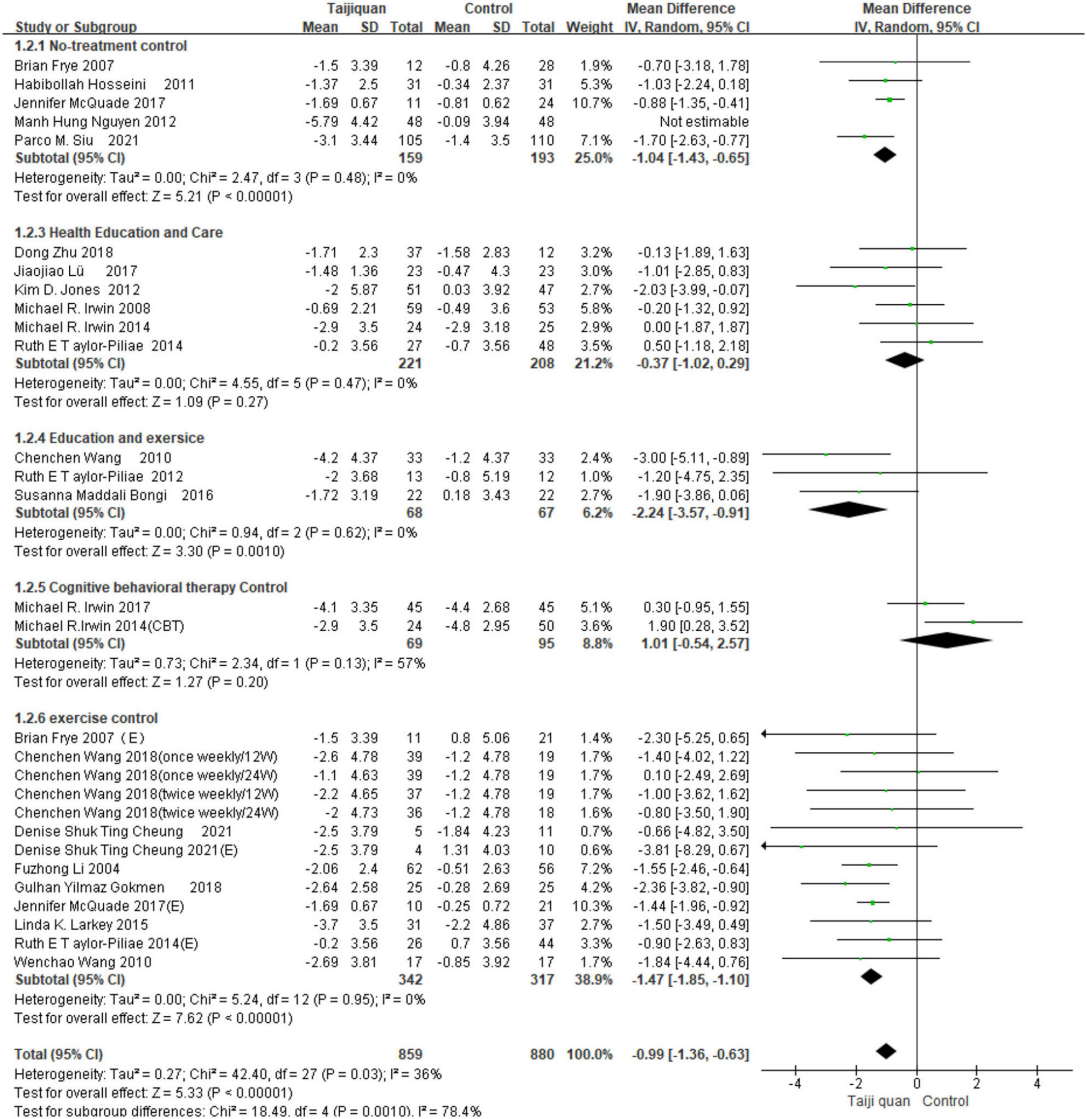
Subgroups of different control groups analyzed forest plot.

**Figure 9:**
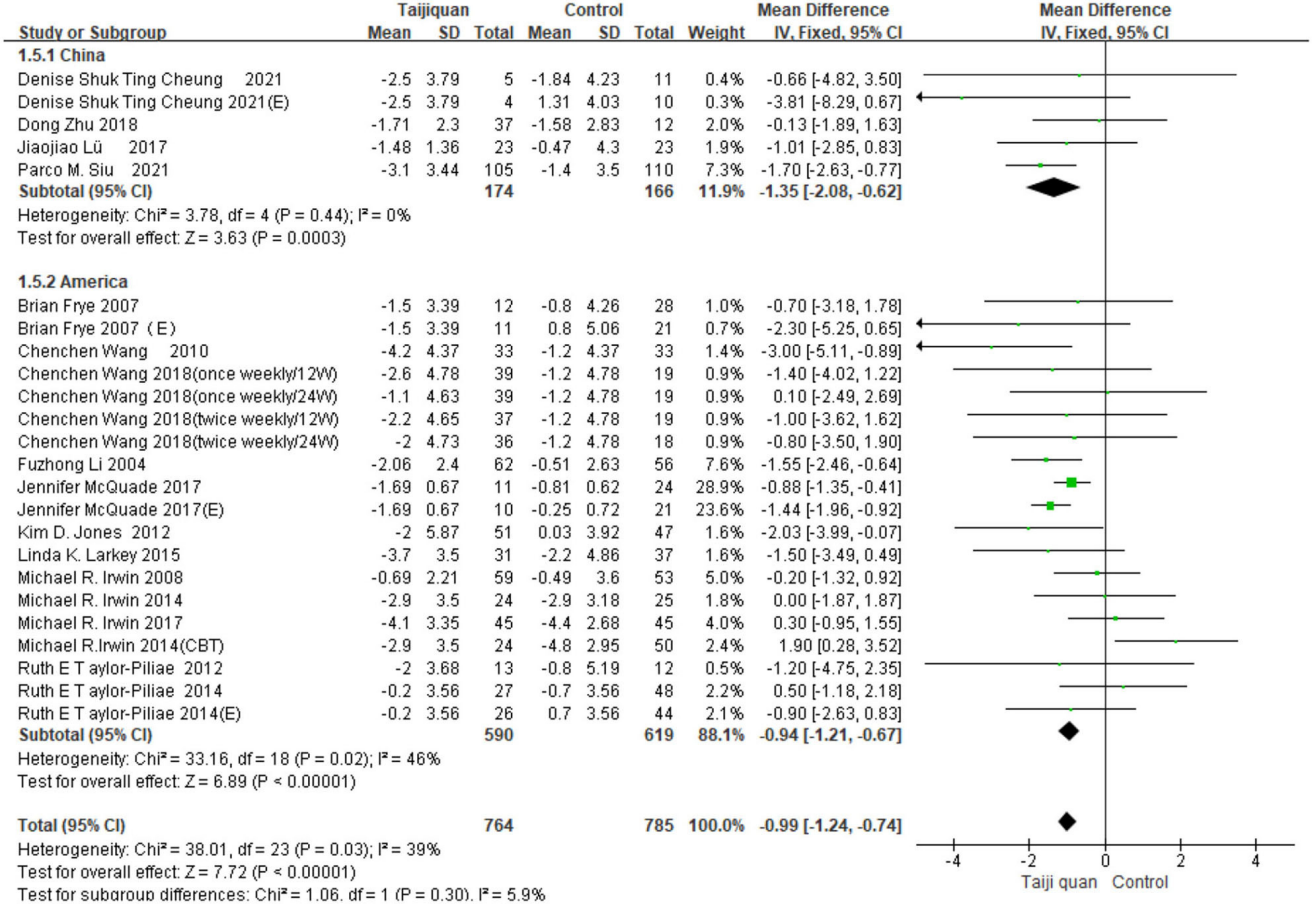
Subgroups of different countries analyzed forest plot.

**Figure 10:**
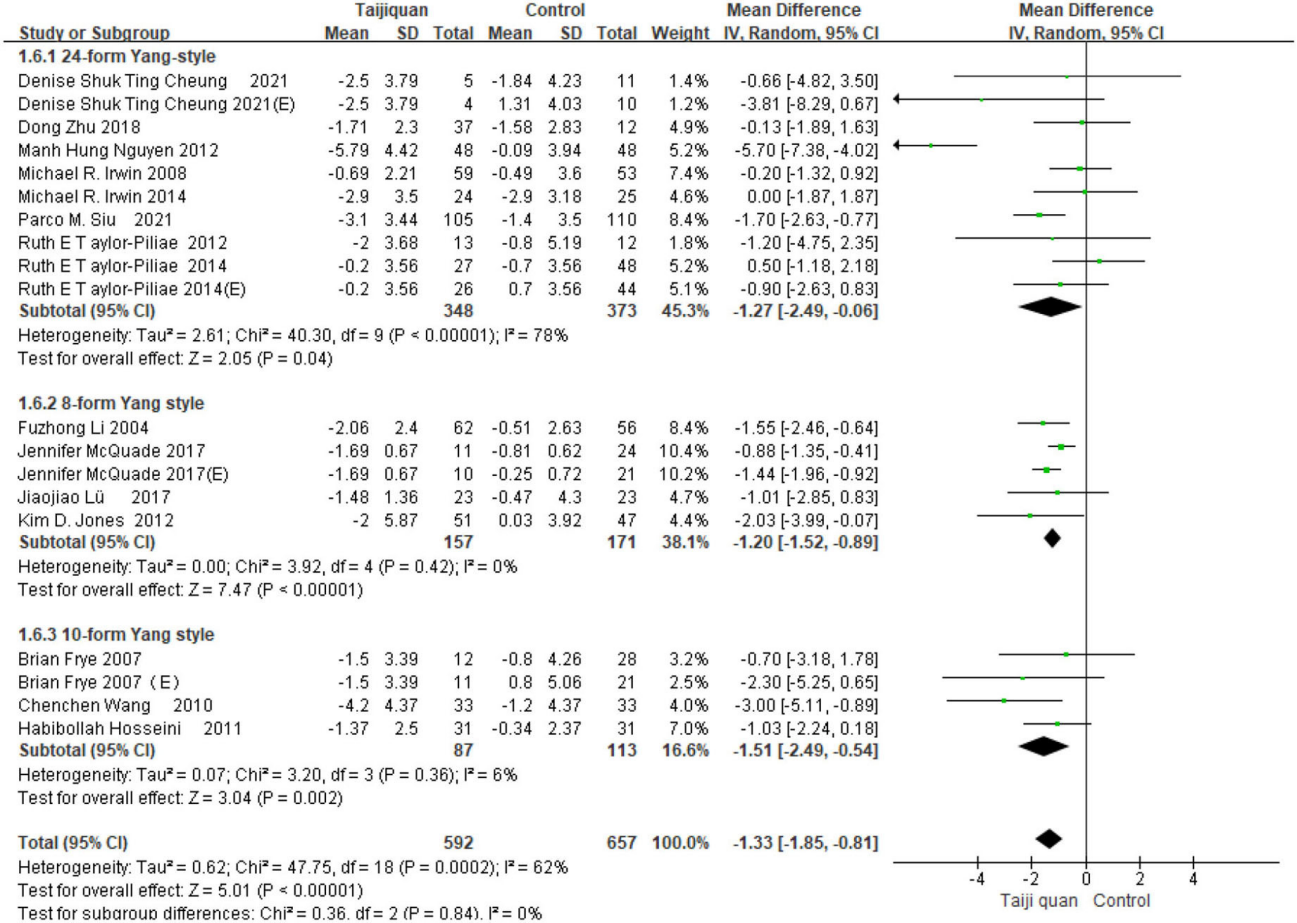
Subgroups of different forms of Taijiquan analyzed forest plot.

**Figure 11:**
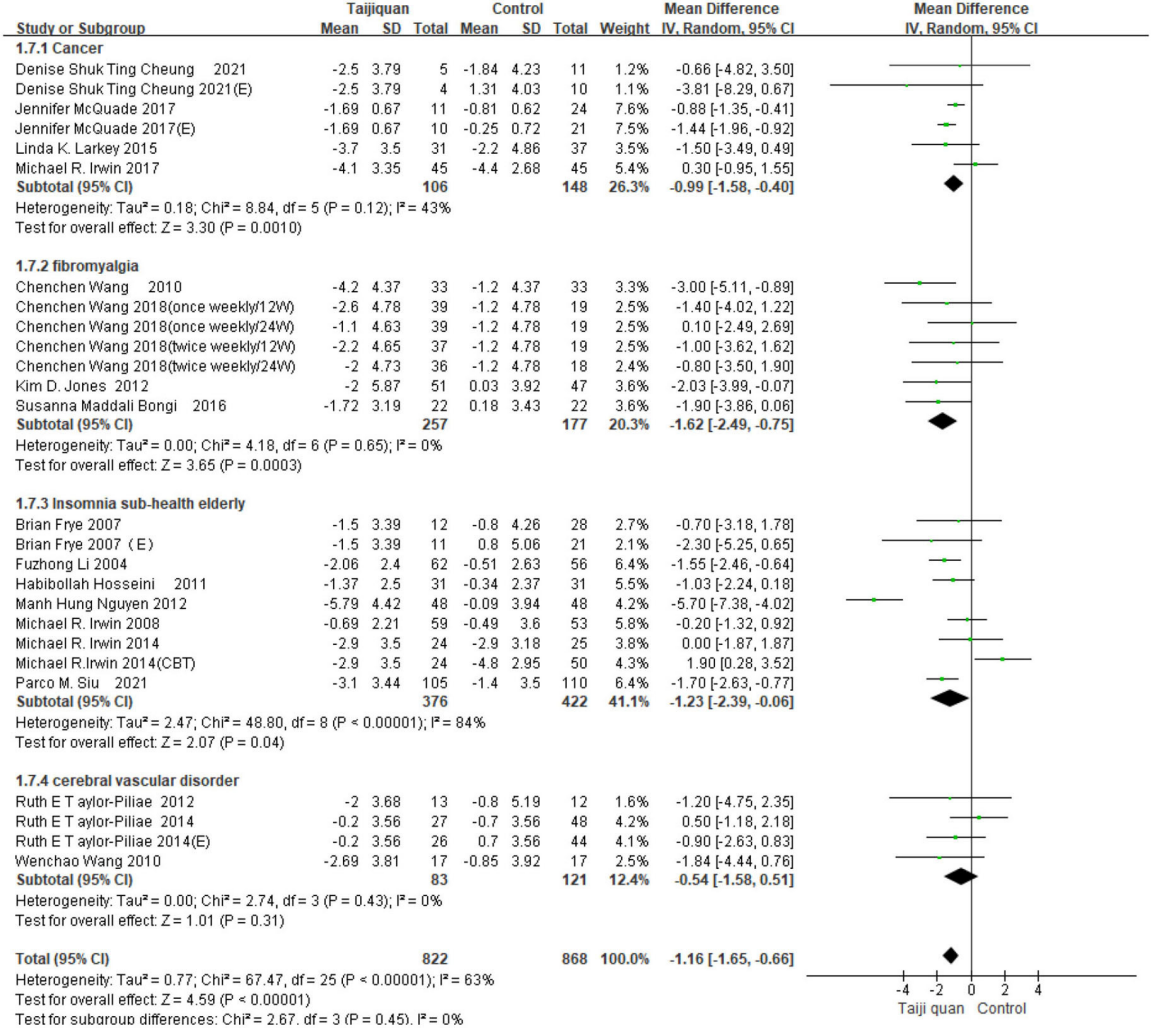
Subgroups of different patients analyzed forest plot.

Five studies showed the effect of Taijiquan on global PSQI scores compared with non-treatment groups. Results of our meta-analysis showed that Taijiquan significantly reduced PSQI scores [MD = −1.95, 95% CI (−3.31, −0.59), *P* = 0.005], and a significant heterogeneity (*I*^2^ = 87%, *P* < 0.00001). When the Nguyen et al. (32) study was excluded, the heterogeneity of the overall PSQI score decreased to 0 (*I*^2^ = 0%, *P* = 0.48), and the meta-analysis still showed a stable, significant effect [MD = −1.04, 95% CI (−1.43, −0.65), *P* < 0.00001] (Figure 8, 1.2.1). Compared with simple exercise control group, Taijiquan has significant difference [MD = −1.47, 95% CI (−1.85, −1.10), *P* < 0.00001] (Figure 8, 1.2.6); When health education was combined with exercise as a control group, Taijiquan continued to significantly improve sleep [MD = −2.24, 95% CI (−3.57, −0.91), *P* = 0.0010] (Figure 8, 1.2.4). In addition, our subgroup analysis also found that compared with simple health education, both Taijiquan and health education or usual care could significantly reduce PSQI scores, but there was no significant difference between them [MD = −0.37, 95% CI (−1.02, 0.29), *P* = 0.27] (Figure 8, 1.2.3). Both cognitive behavioral therapy and Taijiquan can significantly reduce PSQI scores, but there is no significant difference between the two in improving insomnia [MD = 1.01, 95% CI (−0.54, 2.57), *P* = 0.20] (Figure 8, 1.2.5). There was no significant difference between studies in China and the United States, and Taijiquan was equally effective for Americans and Chinese (*P* = 0.30, Figure 9). Current studies mainly focus on Yang's Taijiquan, and different forms of Taijiquan have no significant difference in improving PSQI (*P* = 0.76, Figure 10); Through subgroup analysis of different patients, we found that Taijiquan significantly improved cancer, fibromyalgia, and insomnia of normal elderly people, while there seemed to be no significant difference in patients with cerebrovascular diseases (*P* = 0.31, Figure 11).

## Discussion

In this systematic review and meta-analysis, we identified 21 RCTs of 2022 individuals from seven countries. Overall, taijiquan has a better effect on insomnia improvement compared with exercise and health education alone, and subgroup analyses found that tai chi was as effective as cognitive behavioral therapy in improving insomnia. In terms of different diseases, we found that Taijiquan significantly improved cancer, fibromyalgia, and insomnia in normal elderly people, while there seemed to be no significant difference in patients with cerebrovascular disease. Compared with other studies, Taijiquan was equally effective for Americans and Chinese. There are no influences that have a significant effect on Asians. This systematic evaluation includes all the studies on Taijiquan intervention for insomnia in both Chinese and English, and the research objects include both healthy people and various patients, the old and the young, which is a relatively complete systematic evaluation of Taijiquan intervention for insomnia. Additionally, since no adverse events were reported, Taijiquan could be promoted as a safe intervention for improving sleep quality.

Insomnia is a common problem, with about a quarter of adults suffering from insomnia. Body and mind exercise is a hot topic in the intervention treatment of insomnia. Taijiquan was gradually formed in the late Ming and early Qing Dynasties. It is a centuries-old martial art that combines physical movement and relaxation and is a traditional Chinese sport that has spread worldwide in recent years, more than five million people practice Tai Chi in the US alone (39). At present, Tai Chi has been widely used in the intervention of various diseases, including the rehabilitation of motor function after stroke (40), Parkinson's disease (41), atherosclerosis (42), diabetic foot (43), and so on. The first research on the intervention of taijiquan in insomnia occurred in 2002 when Lu et al. (44) found that taijiquan could improve the sleep of cancer patients. So why does taijiquan affect people's sleep?

The exact biological mechanism of Taijiquan in treating insomnia is not clear, Compared to simple exercise training, current hypotheses suggest that Taijiquan may improve sleep outcomes by reducing sympathetic activity and stimulating the parasympathetic nervous system, restoring the homeostasis balance of sympathetic/parasympathetic function (45). As a low-intensity aerobic exercise, Taijiquan may also encourage the brain to induce normal sleep by inhibiting the non-5-HT spinal system or improving the plasma concentration of pro-inflammatory cytokines to prevent insomnia (46, 47). In addition, studies have shown that Tai Chi can enhance functional connections in the brain (48). It can cause a change in the central nervous medium, thus improving people's sleep conditions and treating insomnia symptoms (46). Studies have shown that chronic insomniacs have reduced hippocampal volume and orbitofrontal gray matter concentration and increased anterior cingulate rostral cortex volume compared to non-insomniacs (15, 49, 50). Taijiquan may improve insomnia by inducing changes in hippocampal volume and orbitofrontal gray matter concentration (51, 52). Changes in brain regions associated with insomnia have been observed in Taijiquan trained individuals. Decreased anterior cingulate cortex homogeneity was observed in patients with long-term Taijiquan use (53). Long-term Taijiquan exercise can slow down gray matter atrophy and this can decrease sympathetic activity (54, 55). In addition, Taijiquan significantly increased functional connectivity between the medial prefrontal cortex and the medial temporal lobe in insomniacs (56). Taijiquan can improve emotional stability and, to some extent, improve sleep quality. The Chinese believe that taijiquan exercises emphasize the leading role of consciousness of “mind first behind body” and pay attention to the two characteristics of “peace of mind” and “relaxation of the body.” “Peace of mind” is to eliminate all the adverse effects of sub-stimuli on the cerebral cortex. The combination of consciousness and exercise can stimulate the cerebral cortex, causing excitement in one area of the cerebral cortex, while other areas enter a state of inhibition and get sufficient rest. Taijiquan can improve the function of the central nervous system, improve the coordination between organs in the body, and play a role in regulating and training brain function (57). Compared with simple exercise training, Taijiquan exercise is to improve people's psychology, life and behavior, adjust the physical and mental conditions of insomnia patients, fundamentally cure insomnia.

This systematic review has some advantages and limitations. We conducted a relatively comprehensive systematic review, and the patient types included cancer, fibromyalgia and normal elderly people, etc. There were many diseases, so we could objectively evaluate the efficacy of Taijiquan on insomnia. Compared with previous systematic reviews (58), we included more RCTs with a larger sample size and strictly limited inclusion and exclusion criteria, with higher credibility. In terms of limitations, we only selected PSQI as the only evaluation index. However, most of the other evaluation results were insufficient or original data could not be obtained. In addition, subgroup analysis is lacking randomized controlled studies for some diseases, which may be biased.

In reviewing almost all about Taijiquan to improve insomnia, the high quality of research, we believe that Taijiquan to improve insomnia is convincing, however, the effects of different groups for different styles of Taijiquan treatment is not clear, Taijiquan differences between different age also needs to continue to study, different intervention dose and frequency of the need to continue to study. In addition, further research is needed on the physiological mechanism of Taijiquan in improving insomnia. Future research directions require more large-sample, multi-center, high-quality randomized controlled trials. It is necessary to further optimize the application of different types of Taijiquan in insomnia.

## Conclusion

In conclusion, our meta-analysis showed that Taijiquan can improve sleep quality and improve insomnia. It provides evidence for taijiquan to treat different insomnia people and improve sleep quality. Subgroup analysis showed that Taijiquan can also improve insomnia caused by different diseases (except cerebrovascular diseases), which can be used as a supplement and alternative treatment for insomnia. There was no difference in the efficacy of different forms of Taijiquan, and there was no significant difference in the effect of Taijiquan on improving sleep between Chinese and Americans.

## Data availability statement

The original contributions presented in the study are included in the article/Supplementary material, further inquiries can be directed to the corresponding author/s.

## Author contributions

DH and ZL conceptualized the study design, search and filter the title and abstract of the article, and confirmed the data and statistical analysis. YW and XW drafted and supplemented the methodology. HL and YH solved the difference. YH provided the funds. YC and XL modified their English and provided help in data analysis. JQ and JC are responsible for supervision and quality control. All authors provided information about the direction of research and the contents of the manuscript. All authors approved the final version of the manuscript.

## Conflict of interest

The authors declare that the research was conducted in the absence of any commercial or financial relationships that could be construed as a potential conflict of interest.

## Publisher's note

All claims expressed in this article are solely those of the authors and do not necessarily represent those of their affiliated organizations, or those of the publisher, the editors and the reviewers. Any product that may be evaluated in this article, or claim that may be made by its manufacturer, is not guaranteed or endorsed by the publisher.
