# Supplementary material for: Effectiveness of Taijiquan in treating insomnia: A systematic review and meta-analysis of randomized controlled studies
Source: Front Psychiatry. 2022 Sep 27;13:892453. doi: 10.3389/fpsyt.2022.892453 (PMC9551091; doi:10.3389/fpsyt.2022.892453)
Supplement: Supplementary file 1 [file Data_Sheet_1.PDF]

Pubmed/(MEDLINE)

#1. "insomnia" OR "sleep disorders" OR "sleep disturbances" OR "sleep initiation" OR "sleep maintenance insomnia" OR "Sleep Initiation and Maintenance Disorders" 57,230 results

#2. "Tai Ji" OR "Tai-ji" OR "Tai Chi" OR "Taijiquan" OR "Tai Chi Chuan" OR "T'ai Chi" OR "Quan, Tai Ji" 2,389 results

#3. #1AND#2 69 results

Table 1 | The specific search strategy of the Pubmed database.

No.	Search items
1	"insomnia" [Title/Abstract]
2	"sleep disorders" [Title/Abstract]
3	"sleep disturbances" [Title/Abstract]
4	"sleep initiation" [Title/Abstract]
5	"sleep maintenance insomnia" [Title/Abstract]
6	"Sleep Initiation and Maintenance Disorders" [Title/Abstract]
7	1 or 2 or 3 or 4 or 5 or 6
8	"Tai Ji"[Title/Abstract]
9	"Tai-ji" [Title/Abstract]
10	"Tai Chi" [Title/Abstract]
11	"Taijiquan" [Title/Abstract]
12	"Tai Chi Chuan" [Title/Abstract]
13	"T'ai Chi" [Title/Abstract]
14	"Quan, Tai Ji" [Title/Abstract]
15	8 or 9 or 10 or 11 or 12 or 13 or 14
16	7 and 15

Embase

1. "insomnia" OR "sleep disorders" OR "sleep disturbances" OR "sleep initiation" OR "sleep maintenance insomnia" OR "Sleep Initiation and Maintenance Disorders" 321948 results

2. "Tai Ji" OR "Tai-ji" OR "Tai Chi" OR "Taijiquan" OR "Tai Chi Chuan" OR "T'ai Chi" OR "Quan, Tai Ji" 4322 results

#1. 1AND2 254 results

The specific search strategy of the Embase database.

No.	Search items
1	“insomnia” [exp]
2	“sleep disorders” [exp]
3	“sleep disturbances” [exp]
4	“sleep initiation” [exp]
5	“sleep maintenance” [exp]
6	“Sleep Initiation and Maintenance Disorders”[exp]
7	1 or 2 or 3 or 4 or 5 or 6
8	“Tai Ji”[exp]
9	“Tai-ji”[exp]
10	“Tai Chi” [exp]
11	“Taijiquan” [exp]
12	“Tai Chi Chuan”[exp]
13	“T'ai Chi” [exp]
14	“Quan, Tai Ji”[exp]
15	8 or 9 or 10 or 11 or 12 or 13 or 14
16	7 and 15

Cochrane

Advanced Search

Title Abstract Keyword:“insomnia” OR “sleep disorders” OR “sleep disturbances” OR “sleep initiation” OR “sleep maintenance insomnia” OR “Sleep Initiation and Maintenance Disorders”

AND Title Abstract Keyword: “Tai Ji” OR “Tai-ji” OR “Tai Chi” OR “Taijiquan” OR “Tai Chi Chuan” OR “T'ai Chi” OR “Quan, Tai Ji”

Run search

73 results

The specific search strategy of the Cochrane database.

No.	Search items
1	“insomnia” [Title Abstract Keyword]
2	“sleep disorders” [Title Abstract Keyword]
3	“sleep disturbances” [Title Abstract Keyword]
4	“sleep initiation” [Title Abstract Keyword]

5	“sleep maintenance” [Title Abstract Keyword]
6	“Sleep Initiation and Maintenance Disorders” [Title Abstract Keyword]
7	1 or 2 or 3 or 4 or 5 or 6
8	“Tai Ji”[Title Abstract Keyword]
9	“Tai-ji” [Title Abstract Keyword]
10	“Tai Chi” [Title Abstract Keyword]
11	“Taijiquan” [Title Abstract Keyword]
12	“Tai Chi Chuan” [Title Abstract Keyword]
13	“T'ai Chi” [Title Abstract Keyword]
14	“Quan, Tai Ji” [Title Abstract Keyword]
15	8 or 9 or 10 or 11 or 12 or 13 or 14
16	7 and 15

Web of Science

# 1 33321 results

TS=(“insomnia” OR “sleep disorders” OR “sleep disturbances” OR “sleep initiation” OR “sleep maintenance insomnia” OR “Sleep Initiation and Maintenance Disorders”)

# 2 2512 results

TS=(“Tai Ji” OR “Tai-ji” OR “Tai Chi” OR “Taijiquan” OR “Tai Chi Chuan” OR “T'ai Chi” OR “Quan, Tai Ji”)

# 3 115 results

#2 AND #1

The specific search strategy of the Web of Science database.

No.	Search items
1	“insomnia” [TS]
2	“sleep disorders” [TS]
3	“sleep disturbances” [TS]
4	“sleep initiation” [TS]
5	“sleep maintenance” [TS]
6	“Sleep Initiation and Maintenance Disorders” [TS]
7	1 or 2 or 3 or 4 or 5 or 6
8	“Tai Ji”[TS]

9	“Tai-ji” [TS]
10	“Tai Chi” [TS]
11	“Taijiquan” [TS]
12	“Tai Chi Chuan” [TS]
13	“T'ai Chi” [TS]
14	“Quan, Tai Ji” [TS]
15	8 or 9 or 10 or 11 or 12 or 13 or 14
16	7 and 15

CNKI

# 1 39600 results

主题=太极拳 + 太极

# 2 60700 results

主题=睡眠障碍 + 睡眠困难 + 失眠 + 不寐

# 3 129 results

#2 AND #1

The specific search strategy of the CNKI database.

No.	Search items
1	“太极拳” [主题]
2	“太极” [主题]
3	1 or 2
4	“睡眠障碍”[主题]
5	“睡眠困难” [主题]
6	“失眠” [主题]
7	“不寐” [主题]
8	4 or 5 or 6 or 7
9	随机对照试验 [全文]
10	RCT [全文]

11	9 or 10
12	3 and 8 and 11

WANGFANG

# 1　39202 results

主题=太极拳 OR 太极

# 2　110905 results

主题=睡眠障碍 OR 睡眠困难 OR 失眠 OR 不寐

# 3　#2 AND #1

169 results

The specific search strategy of the WANGFANG database.

No.	Search items
1	“太极拳” [主题]
2	“太极” [主题]
3	1 or 2
4	“睡眠障碍”[主题]
5	“睡眠困难” [主题]
6	“失眠” [主题]
7	“不寐” [主题]
8	4 or 5 or 6 or 7
9	随机对照试验 [全文]
10	RCT [全文]
11	9 or 10
12	3 and 8 and 11

VIP

# 1　11054 results

U=太极拳 OR 太极 OR tai ji quan OR taijiquan

# 2　57780 results

U=睡眠障碍 OR sleep disorder OR sleep disorders OR 睡眠困难 OR 失眠 OR insomnia OR sleeplessness OR 不寐

# 3　55 results

#2 AND #1

The specific search strategy of the VIP database.

No.	Search items
1	“太极拳” [U]
2	“太极” [U]
3	“tai ji quan” [U]
4	“taijiquan”[U]
5	1 or 2 or 3 or4
6	“sleep disorder” [U]
7	“sleep disorders”[U]
8	“insomnia”[U]
9	“ sleeplessness”[U]
10	“睡眠障碍”[U]
11	“睡眠困难” [U]
12	“失眠” [U]
13	“不寐” [U]
14	6 or 7 or 8 or 9 or 10 or 11 or 12 or 13
15	5 and 14

SinoMed
# 1 1500 results
("太极拳"[加权:扩展] OR "太极"[加权:扩展])
# 2 52699 results
("睡眠障碍"[加权:扩展] OR "睡眠困难"[加权:扩展] OR "失眠"[加权:扩展] OR "不寐"[加权:扩展])
# 3 #2 AND #1
42 **results**

The specific search strategy of the SinoMed database.

No.	Search items
1	“太极拳”[主题]
2	“太极”[主题]
3	1 or 2

4	“睡眠障碍”[主题]
5	“睡眠困难” [主题]
6	“失眠” [主题]
7	“不寐”[主题]
8	4 or 5 or 6 or 7
9	3 and 8
